# Impaired cognitive function in Crohn’s disease: Relationship to disease activity

**DOI:** 10.1016/j.bbih.2020.100093

**Published:** 2020-06-08

**Authors:** Gerard Clarke, Paul J. Kennedy, John A. Groeger, Eamonn MM. Quigley, Fergus Shanahan, John F. Cryan, Timothy G. Dinan

**Affiliations:** a APC Microbiome Ireland, University College Cork, Cork, Ireland; b Department of Psychiatry and Neurobehavioural Science, University College Cork, Cork, Ireland; c School of Applied Psychology, University College Cork, Cork, Ireland; d Department of Medicine, University College Cork, Cork, Ireland; e Department of Anatomy and Neuroscience, University College Cork, Cork, Ireland

**Keywords:** Cognition, Gut-brain axis, Inflammatory bowel disease, Crohn’s disease, Tryptophan, Immune system, IBD, Inflammatory bowel disease, CD, Crohn’s disease, UC, ulcerative colitis, CAR, cortisol awakening response, BMI, body mass index, PAL, Paired Associates Learning, CANTAB®, Cambridge Neuropsychological Test Automated Battery, IED, Intra-Extradimensional Set Shift, SWM, Spatial Working Memory, ACC, anterior cingulate cortex, MRT, mean response time, PFC, prefrontal cortex, MCAR, missing completely at random, EM, Expectation-maximization, HBI, Harvey Bradshaw Index, SCCAI, Short Clinical Colitis Activity Index, HSD, Honestly Significant Difference

## Abstract

**Background & aims:**

Impaired attention and response inhibition have been reported in patients with Crohn’s disease (CD) in clinical remission. Prospective studies are needed to determine whether this is a stable feature of CD and whether a similar impairment is evident in ulcerative colitis (UC). Thus, our aims were to examine whether patients with CD and UC exhibited a persistent impairment in attentional performance, and if this impairment was related to key biological indices of relevance to cognition.

**Methods:**

A prospective observational study was conducted on fifteen patients with CD and 7 with UC in clinical remission recruited from a specialty clinic and 30 healthy matched control participants. A neuropsychological assessment was carried out at baseline (visit 1) and at a 6 month follow-up (visit 2). Plasma proinflammatory cytokines, the plasma kynurenine:tryptophan (Kyn:Trp) ratio and the salivary cortisol awakening response (CAR) were also determined at each visit.

**Results:**

Across visits, patients with CD exhibited impaired attentional performance (*p ​=* 0.023). Plasma IL-6 (*P* ​= ​0.001) and the Kyn:Trp ratio (*P* ​= ​0.03) were consistently elevated and the CAR significantly blunted (*P* ​< ​0.05) in patients with CD. No significant relationships were identified between any biochemical parameter and altered cognitive performance.

**Conclusions:**

Impaired cognitive function is a stable feature of patients with CD. These data suggest that even where remission has been achieved, the functional impact of an organic gastrointestinal disorder on cognition is still evident. However, it is unclear at present if physiological changes due to disease activity play a role in cognitive impairment in CD.

## Highlights

• Crohn’s disease (CD) patients previously associated with impaired cognition.
• CD patients consistently exhibited impaired attentional performance at multiple visits.
• Functional impact of organic gastrointestinal disorder on cognition still evident in remission.
• Increased IL-6, kynurenine pathway activation and blunted cortisol awakening response in CD.
• Biochemical parameters not associated with altered cognitive performance.

## 1 Introduction

Over recent years the impact of psychosocial and psychological factors, via pathways along the brain-gut axis in inflammatory bowel disease (IBD), have become increasingly recognized (Bonaz and Bernstein, 2013; Gracie et al., 2018; Rhee et al., 2009). For example, it has been reported that perceived stress, negative mood and major life events are more predictive of a symptomatic flare-up of IBD than the use of either non-steroidal anti-inflammatory drugsor antibiotics, and non-enteric infections (Bernstein et al., 2010). Pre-clinical evidence further suggests that stress may be involved in the initiation, as well as the relapse, of symptoms in IBD (Qiu et al., 1999). Pathogenic processes such as chronic inflammation may exert a significant influence on normal brain-gut communications in IBD (Cryan et al., 2019; Dantzer, 2004) and it is clear that a pro-inflammatory phenotype significantly increases the risk of depression and anxiety related symptoms, both of which are commonly found in patients with IBD (Goodhand et al., 2012). The relationship between IBD disease activity and psychological disorders is likely bidirectional (Gracie et al., 2018).

In a recent cross-sectional study, patients with Crohn’s disease (CD), who were in remission at the time of testing, were found to exhibit impaired attentional performance on a battery of neuropsychological tests (Kennedy et al., 2014). Prior to this investigation a limited number of cognitive assessments had been reported in IBD, with the most consistent finding being reduced verbal IQ performance (Berrill et al., 2013; Dancey et al., 2009). More recently, impaired neurocognitive and psychomotor function across areas of convergent thinking, perceptive abilities, sophisticated operative thinking, processing speed, verbal learning, and delayed recall memory (Tadin Hadjina et al., 2019; Whitehouse et al., 2019) as well as cognitive inflexibility have been reported (Petruo et al., 2017). Attention and memory impairments have also been reported in pediatric patients with IBD (Piasecki et al., 2017).

Nevertheless, and the despite the important association between illness cognitions and quality of life (Gurkova and Soosova, 2018), there are a paucity of investigations into cognitive performance in IBD and it remains a largely overlooked aspect of the disease. Furthermore, the nature of the biological processes mediating altered cognitive function in patients in apparent remission are currently unknown although alterations in brain white matter microstructural properties (Hou et al., 2020) and functional disruption in the anterior cingulate cortex (ACC) and the right inferior frontal gyrus have been noted (Petruo et al., 2017). Inflammation, stress and the stress- and immune-mediated changes in the metabolism of tryptophan along the kynurenine pathway are potential mechanisms through which CNS function could be modulated (Kennedy et al., 2012).

In this study, we carried out a prospective assessment of cognitive performance in patients with both CD and UC in clinical remission at each study visit in comparison to matched healthy control participants (Kennedy et al., 2014). Based on our prior findings, our *a priori* hypothesis was that these patients would display a consistent impairment on a test of attentional performance. This was our primary outcome measure, with visuospatial episodic memory as the main secondary endpoint. We also tested the hypothesis that any impairments observed were related to altered brain-gut axis signaling, due to the influence of systemic inflammation, hypothalamic-pituitary-adrenal (HPA)-axis dysfunction and related changes in metabolism of tryptophan along the kynurenine pathway.

## 2 Methods

### 2.1 Study population

Participants from our preliminary investigation (Kennedy et al., 2014) were re-enrolled for two follow-up assessments which were designated as visit 1 and visit 2. Only IBD patients who were in remission and remained in remission throughout the study were included. Remission was defined as a Harvey Bradshaw Index (HBI) score <5 for CD (Cuffari et al., 2001) and a Short Clinical Colitis Activity Index (SCCAI) score ​≤ ​3 for UC (Walmsley et al., 1998). Patients were recruited from a specialty clinic at Cork University Hospital. Healthy control participants were recruited via advertisement from the staff and student population of University College Cork. Study participants were males and femails between 18 and 50 years of age. Exclusion criteria included use of psychoactive medications (including anxiolytics, antipsychotics, antidepressants, and opioid based pain relievers), corticosteroid use in the prior 4 weeks (budesonide, which has lower systemic bioavailability, was allowed at time of testing), antibiotic use within the prior four weeks, history of alcohol abuse and recent (within 6 months) abdominal surgery.

### 2.2 Study procedures: Visit 1 and visit 2

The study protocol (APC024 2010) and all procedures were approved by the University College Cork Clinical Research Ethics Committee of The Cork Teaching Hospitals and conducted in accordance with the ICH Guidelines on Good Clinical Practice, and the Declaration of Helsinki. Study participants meeting inclusion criteria provided written informed consent prior to any study procedures. A total of 52 participants were assessed at visit 1 and 47 returned for 6 month follow-up at visit 2. A maximum time window of plus or minus 4 weeks of a scheduled follow-up visit date was permitted. At baseline, groups were matched on the basis of age, verbal IQ and body mass index (BMI). At each visit, medical history and a brief medical examination were carried out by an experienced clinical research nurse who recorded participants’ vital signs, BMI, noted all medications used by patients, and collected a venous blood sample for research purposes (see below) and for assessment of full blood count, renal function, serum electrolytes and liver enzymes. Clinically significant abnormalities in these latter blood tests at visit 1 or 2 led to exclusion from the study.

### 2.3 Measures

#### 2.3.1 Anxiety & depression

Symptoms of anxiety and depression were assessed at visit 1 and 2 using the self-reported Hospital Anxiety and Depression Scale (HADS; (Zigmond and Snaith, 1983)) and the Patient Health Questionnaire (PHQ-9; (Kroenke et al., 2001)).

#### 2.3.2 Proinflammatory cytokine sampling & analysis

At each visit, 10 ​ml of whole blood was collected in EDTA tubes. Samples were centrifuged immediately at 1000×*g* for 15 ​min and aliquoted plasma samples were frozen at −80 ​°C until analysis. Plasma levels of IL-6, IL-8, and TNF-α were assayed in duplicate using a high sensitivity commercially available electrochemiluminescence MULTI-SPOT® Meso Scale Discovery kit (MSD, Rockville, MD, USA) as per the manufacturer’s instructions. The median lower limits of detection for each cytokine are; IL-6- 0.06 ​pg/ml, IL-8- 0.04 ​pg/ml, TNF-α- 0.04 ​pg/ml.

#### 2.3.3 Kynurenine and tryptophan analysis

Tryptophan and kynurenine pathway metabolites were determined as previously described (Clarke et al., 2009). Plasma samples were spiked with internal standard (3-Nitro l-tyrosine) prior to being deproteinised by the addition of 20 ​μl of 4M perchloric acid to 200 ​μl of sample. Samples were centrifuged at 21000 ​g on a Hettich Mikro 22R centrifuge (AGB, Dublin, Ireland) for 20 ​min at 4 ​°C and 100 ​μl of supernatant transferred to a HPLC vial for analysis on the HPLC system (UV and FLD detection). All samples were injected onto a reversed phase Luna 3 ​μm C18 (2) 150 ​× ​2 ​mm column (Phenomenex), which was protected by Krudkatcher disposable pre-column filters (Phenomenex) and SecurityGuard cartridges (Phenomenex). The mobile phase consisted of 50 ​mM acetic acid, 100 ​mM zinc acetate with 3% (v/v) acetonitrile and was filtered through Millipore 0.45 ​μm HV Durapore membrane filters (AGB) and vacuum degassed prior to use. Compounds were eluted isocratically over a 30-min runtime at a flow rate of 0.3 ​mls/min after a 20 ​μl injection. The column was maintained at a temperature of 30 ​°C and samples/standards were kept at 8 ​°C in the cooled autoinjector prior to injection. The fluorescent detector was set at an excitation wavelength of 254 ​nm and an emission wavelength of 404 ​nm. The UV detector was set to 330 ​nm. L-tryptophan and kynurenine were identified by their characteristic retention times as determined by standard injections which were run at regular intervals during the sample analysis. Analyte: Internal standard peak height rations were measured and compared with standard injections and results were expressed as ng/ml of plasma.

#### 2.3.4 Salivary cortisol analysis

HPA axis function was determined by measuring the salivary cortisol awakening response (CAR) as previously described (Kennedy et al., 2014). Saliva samples were stored at −80 °C until analysis. Cortisol concentrations were determined using the Cortisol Enzyme Immunoassay Kit as per manufacturers’ instruction (Enzo®, Life Sciences). Assay detection limit was 0.16 nmol/L. Inter and intra assay % C.Vs were 8.7% and 7.3% respectively.

#### 2.3.5 Cognitive assessments

At visit 1 and 2, participants completed a computerized Stroop test (Xavier Educational Software Ltd, Bangor, Wales), and the Paired Associates Learning (PAL) test from the Cambridge Neuropsychological Test Automated Battery (CANTAB®; Cambridge Cognition, LTD (Robbins and Sahakian, 1994)). To confirm our preliminary finding that other cognitive domains were not affected, participants were also assessed using the Intra-Extradimensional Set Shift (IED) and Spatial Working Memory (SWM) tests from the CANTAB®. Parallel versions of the PAL and IED test were used at visit 1 and 2 to reduce practice effects. Parallel versions of the SWM or Stroop tests are not available and the same versions were used at each visit. The cognitive assessment lasted approximately 45 min with each participant first completing the Big/Little Circle as a short familiarization task, followed by the IED, PAL and SWM tests from the CANTAB®, and finally the Stroop test. All assessments at visit 1 and 2 were conducted by a trained administrator who issued standardized verbal instructions to participants on the use of a portable touch screen Sahara i440D Slate Tablet PC (Sand Dune Ventures, Tablet Kiosk). A measure of pre-morbid IQ was obtained using the National Adult Reading Test-2 (NART-2, (Nelson and Willison, 1991) and converted to Wechsler Adult Intelligence Scale-Revised (WAIS-R) full scale IQ scores**.** Detailed information on CANTAB® tests are available elsewhere (Fray and Robbins, 1996; Sahakian and Owen, 1992) and summarised below..

##### 2.3.5.1 Stroop word color interference test (Stroop test)

The Stroop is an executive function test and specifically measures selective attention and response inhibition. During the interference stage of the Stroop, inhibition of the prepotent response primarily engages the ACC, with general Stroop performance also requiring input from a number of regions of the temporal and parietal lobes (Alvarez and Emory, 2006; Botvinick et al., 2004; Strauss et al., 2006). The computerized Stroop test used in the current study is based on the Victoria Stroop Test as previously described (Assef et al., 2007). Response speed in milliseconds (ms) is recorded on each trial with an overall mean response time (MRT) for each of 3 stages consisting of 24 trials. In stage 1, on each trial participants are required to name a color word printed in the white [word naming]; Stage 2, on each trial participants name the color of a line of stars which appear on screen [color naming]; Stage 3, on each trial participants must name the color a word is printed in, which is incongruent to the word it spells e.g. the word ‘blue’ printed in the color red, [interference stage]). The main outcome measure is the *Stroop effect*, which is calculated by subtracting the MRT of stage 3 [Interference Stage], from the MRT on stage 1 [word naming]. The Stroop effect was measured at visits 1 and 2.

##### 2.3.5.2 Paired Associates Learning (PAL, Parallel mode)

PAL is a visuospatial episodic memory test which assesses new learning, list memory and list learning, and has demonstrated sensitivity to changes in the function of hippocampal brain regions (Blackwell et al., 2004; Owen et al., 1995; Swainson et al., 2001; Sweeney et al., 2000). PAL also engages a number of additional brain regions comprising a fronto-parietal network during encoding phases, and posterior cingulate and left cuneus regions during retrieval stages (de Rover et al., 2011). The main outcome measure was *Total errors* (adjusted) assessed at visit 1 and 2.

##### 2.3.5.3 Intra-Extradimensional Set Shift (IED)

The IED is an executive function test and measures rule acquisition and reversal, attentional set formation, maintenance and shifting (Downes et al., 1989; Sahakian and Owen, 1992). Reversal learning involves ventral prefrontal cortex (PFC) brain regions, while attentional set-shifting engages the dorsolateral PFC (Nagahama et al., 2001). The main outcome measure was *Total errors* (adjusted), assessed at visit 1 and 2.

##### 2.3.5.4 Spatial Working Memory (SWM)

The SWM is a working memory test which involves on-line monitoring and updating of information and self-ordered searching. The SWM has shown sensitivity to frontal lobe dysfunction (Owen et al., 1996; Robbins et al., 1998). The main outcome measure was *Total errors* assessed at visit 1 and 2.

### 2.6 Statistical analysis

Group characteristics (age, IQ and BMI) at visit 1 were analyzed by one-way analysis of variance (ANOVA). Chi-square (χ^2^) was used to examine gender distribution across groups. Changes in patient disease activity measured using the HBI and SCCAI were assessed using paired samples t-tests between visit 1 and 2. To allow for repeated measures analysis and to avoid bias that may be introduced by using list-wise deletion of incomplete cases (Graham, 2009; Rubin et al., 2007; Twisk and de Vente, 2002), missing data analysis was performed on variables subject to prospective analysis. In total, 5.5% of cognitive performance data and 6.2% of biochemical measure data were missing at visit 2. We first determined that data were missing completely at random (MCAR) using Littles MCAR test ((Little, 1988); χ^2^ (150) ​= ​170.512, p ​= ​0.121)). Single imputation was then performed using the Expectation-maximization (EM) algorithm (Dempster et al., 1977) with complete values at visit 1 as predictor variables to impute missing visit 2 values, as previously described (Guloksuz et al., 2013; Laaksonen et al., 2011; Lintvedt et al., 2013). Following data imputation, normality checks were performed. CANTAB ® variables were not normally distributed and were transformed as follows: IED and PAL outcome variables were normalized using logarithmic base 10 (log10) transformations and SWM outcome variables normalized using square-root transformations. Plasma cytokine & salivary cortisol data were not normally distributed and were normalized using a natural logarithmic transformation (ln). Total cortisol levels across the three collection time points at visit 1 and 2 were determined using an area under the curve with respect to ground (AUCg) analysis (Pruessner et al., 2003). Twenty-eight healthy control participants, 14 patients with CD and 6 patients with UC provided saliva samples as instructed and useable for analysis at Visit 1. One IL-6 sample and one IL-8 sample from two separate patients with UC were out of the detection range and cytokine imputation and repeated measures analysis was conducted with these participants excluded. Univariate repeated measures ANOVA was used to determine group differences across Visit 1 and Visit 2 on the Stroop test, PAL, IED, and SWM, HADS-anxiety (HADS-A), HADS-depression (HADS-D), PHQ-9 and PSQI scores, and for plasma cytokines, tryptophan, kynurenine, the kynurenine:tryptophan (Kyn:Trp) ratio and CAR, followed by inspection of post-hoc Tukey Honestly Significant Difference (HSD) tests where significant main group effects were found. Where Mauchly’s test of sphericity was significant, the Greenhouse-Geisser or Huynh-Feldt correction was applied. Where significant main effects of visit or group by visit interactions were found, planned comparisons of group differences at each visit individually were investigated using one-way ANOVA followed by inspection of post-hoc Tukey HSD tests as appropriate. To determine within group changes on each measure where main effects of visit, or visit by group interactions were found, paired samples t-tests with a Bonferroni correction were carried out within each group. To determine relationships between cognitive performance and biochemical and questionnaire and clinical measures across visits within CD patients, a composite value was calculated (mean of Visit 1 and 2) for the Stroop effect, PAL total errors, IL-6, IL-8, TNF-α, tryptophan, kynurenine, the Kyn:Trp ratio, the CAR, HADS-A, HADS-D, PHQ-9, PSQI, and the HBI. Spearman’s rho was then examined to identify significant relationships between composite cognitive performance, biochemical and clinical measures. Non-transformed data are presented as mean ​± ​standard error of the mean (SEM). Effect sizes are reported as partial Eta squared (η_p_^2^). All statistical procedures were carried out using IBM SPSS Statistics 20.0 for Windows software package.

## 3 Results

### 3.1 Sample characteristics

Group characteristics for healthy control participants, patients with CD and UC are presented in Table 1 and Table 2. Patients with IBD were using the following medications at Visit 1; 6-MP (CD n ​= ​7; UC n ​= ​2), mesalamine (CD n ​= ​3; UC n ​= ​5), adalimumab (CD n ​= ​4), mesalazine (CD n ​= ​2; UC n ​= ​1), azathioprine (CD n ​= ​2; UC n ​= ​1), budesonide (CD n ​= ​2), and sulfasalazine (CD n ​= ​1). Only one change in medication was recorded at visit 2 (budesonide: CD n ​= ​1).

**Table 1:** Comparison of group demographics at Visit 1.

*Baseline Demographics*	Healthy Controls (n ​= ​30)	CD (n ​= ​15)	UC (n ​= ​7)	*P*-value
Age	28.23 ​± ​1.71	31.93 ​± ​2.05	35 ​± ​4.22	.16
Gender: Male (%) Female (%)	10 (33.3%) 20 (66.7%)	11 (73.3%) 4 (26.7%)	2 (28.6%) 5 (71.4%)	.026∗
BMI	23.27 ​± ​.71	25.47 ​± ​.76	23.93 ​± ​1.23	.15
WAIS-R Full Scale IQ (NART conversion)	109.19 ​± ​1.19	103.16 ​± ​2.92	103.11 ​± ​5.16	.069

CD, Crohn’s disease; UC, ulcerative colitis; BMI, body mass index; WAIS-R, Wechsler Adult Intelligence Scale-Revised; NART, National Adult Reading Test;. Data are mean ​± ​S.E.M.

**Table 2:** Summary and group comparisons of mean anxiety, depression, sleep disturbance and disease activity scores at visit 1 & visit 2.

	Healthy Controls (n ​= ​30)		CD (n ​= ​15)		UC (n ​= ​7)		p-value
	Visit 1	Visit 2	Visit 1	Visit 2	Visit 1	Visit 2	
HADS-A	3.6 ​± ​.53	3.7 ​± ​.72	5.6 ​± ​.86	5 ​± ​.85	6.86 ​± ​1.94	5.33 ​± ​1.45	0.185
HADS-D	1.43 ​± ​.32	1.15 ​± ​.36	3.2 ​± ​.99	2.46 ​± ​.86	3.57 ​± ​1.44	3.5 ​± ​1.12	0.029
PHQ-9	1.37 ​± ​.39	1.26 ​± ​.45	3.33 ​± ​1.14	2.92 ​± ​1.07	2.28 ​± ​.92	2.83 ​± ​1.19	
HBI Total Score (Remission ​< ​5)	–	–	1.57 ​± ​0.35	2.12 ​± ​0.51	–	–	.216#
SCCAI (Remission ​≤ ​3)	–	–	–	–	1.43 ​± ​0.37	2.33 ​± ​0.33	.058#

HADS-A/D, Hospital Anxiety and Depression Scale- Anxiety/Depression; PHQ-9, Patient Health Questionnaire; PSQI, Pittsburgh Sleep Quality Index; HBI, Harvey Bradshaw Index; SCCAI, Short Clinical Colitis Activity Index.Data are mean ​± ​S.E.M.

### 3.2 Cognitive performance

#### 3.2.1 Attentional performance (Stroop test) is impaired in Crohn’s disease

**Fig. 1:**
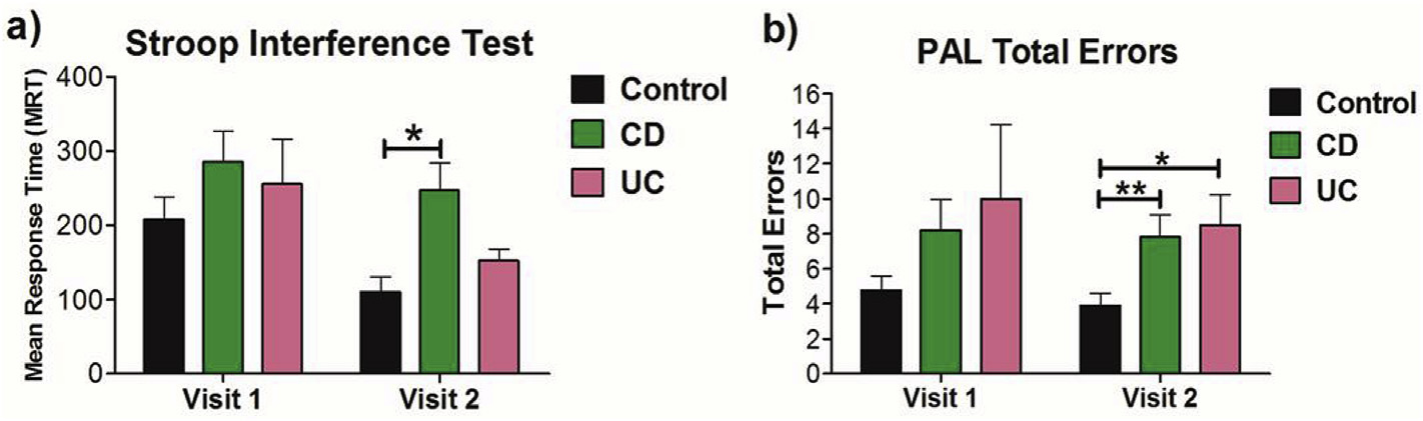
Group comparison of a) selective attention and response inhibition on the Stroop test at Visit 1 and Visit 2; b) Visuospatial memory performance on the Paired Associates Learning (PAL) test at Visit 1 and Visit 2 (∗∗P ​< ​0.01; ∗P ​< ​0.05). CD, Crohn’s disease; UC, ulcerative colitis. Data are presented as mean ​± ​SEM.

Across visits 1 and 2,there was an overall main effect of group (F(2, 49) ​= ​3.824; *P* ​= ​0.029, η_p_^2^ ​= ​0.135) with patients with CD exhibiting impaired attentional performance when compared to healthy controls (*P* ​= ​0.022). Attentional performance in patients with CD was most impaired at visit 2, when compared to healthy control participants (*P* ​= ​0.002, see Fig. 1a), Healthy control participants exhibited a significant learning effect in performance between visit 1 and 2 (*P* ​= ​0.006), whereas patients with CD (*P* ​= ​0.66) or UC (*P* ​= ​0.327), did not.

#### 3.2.2 Visuospatial episodic memory (Paired Associate Learning; PAL) is impaired in patients with Crohn’s disease and ulcerative colitis

Across visits 1 and 2, there was an overall main effect of group on the total number of errors on the PAL test (F (2, 49) ​= ​5.828; *P* ​= ​0.005, η_p_^2^ ​= ​0.192) with patients with CD exhibiting significantly impaired performance the PAL when compared with healthy controls (*P* ​= ​0.013) but not when compared to patients with UC (*P* ​= ​0.984). A greater number of errors made on the PAL test by patients with UC approached significance when compared to healthy controls (*P* ​= ​0.051). Further analysis revealed that visuospatial memory performance was most impaired at visit 2 in both patients with CD (*P* ​= ​0.008) and patients with UC (*P* ​= ​0.019; see Fig. 1b).

#### 3.2.3 Executive function (intra/extra dimensional shift; IED) is not different between patients and healthy controls

No significant group differences were identified across visit 1 and 2 on executive function (all *P* ​> ​0.05; see Table 3).

**Table 3:** Mean test scores and group comparisons of performance on the IED and SWM tests at Visit 1 and Visit 2.

Cognitive Test	Control		CD		UC		P- value
	Baseline	6 ​Months	Baseline	6 ​Months	Baseline	6 ​Months	
IED Total Errors (Adjusted)	22.77 ​± ​6.19	18.02 ​± ​3	16.47 ​± ​3.59	17.56 ​± ​2.91	31.86 ​± ​8.26	17.56 ​± ​2.91	0.086
SWM Total errors	14.27 ​± ​2.77	15.54 ​± ​2.41	18.93 ​± ​5.21	17.65 ​± ​4.82	16.71 ​± ​5	14.17 ​± ​4.48	0.896

*IED, Intra-extra dimensional set shift; SWM, Spatial Working Memory; CD, Crohn’s disease; UC, ulcerative colitis. (P-value ​= ​ANOVA from repeated measures analysis*)*. Data are mean ​± ​S.E.M.*

#### 3.2.4 Spatial Working Memory (SWM) is not different between patients and healthy controls

No significant group differences were identified across Visit 1 and 2 on working memory performance (all *P* ​> ​0.05; see Table 3**)**.

### 3.3 The cortisol awakening response (CAR) is blunted at visit 1 and 2 in patients with Crohn’s disease

**Fig. 2:**
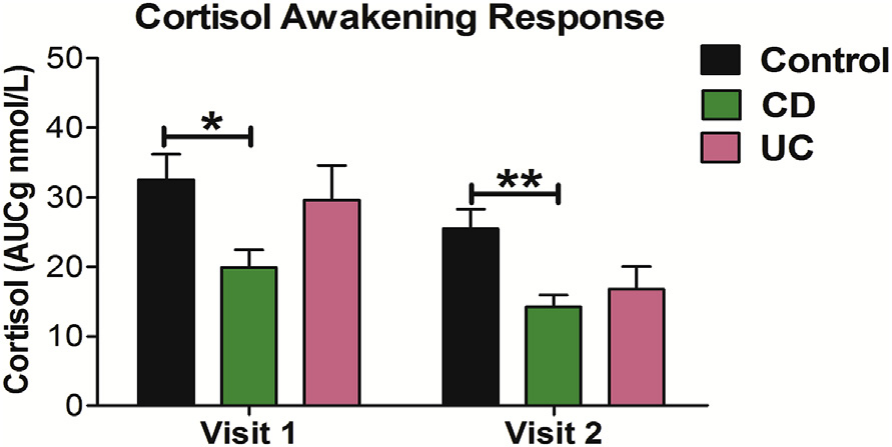
Group comparisons of the cortisol awakening response determined using an area under the curve with respect to ground (AUC_g_) calculation on all three measurement time points (upon wakening, 1 ​h after wakening and 3 ​h after wakening) at Visit 1 and 2 (∗∗P ​< ​0.01, ∗P ​< ​0.05). CD, Crohn’s disease; UC, ulcerative colitis. Data are presented as mean ​± ​SEM.

Across visit 1 and 2 there was a significant main effect of group (F (2, 45) ​= ​7.267; *P* ​= ​0.002, η_p_^2^ ​= ​0.244) in which the cortisol awakening response was significantly blunted in patients with CD when compared to healthy control participants (*P* ​= ​0.001) but not when compared to patients with UC (*P* ​= ​0.426; see Fig. 2**).**

### 3.4 Plasma cytokine levels

#### 3.4.1 IL-6 is elevated at visit 1 and 2 in patients with Crohn’s disease

Across Visit 1 and 2 there a significant main effect of group for plasma levels of IL-6 (F (2, 49) ​= ​9.453; *P* ​< ​0.001, η_p_^2^ ​= ​0.278), with higher levels of IL-6 in patients with CD when compared to healthy controls (*P* ​< ​0.001) but not patients with UC (*P* ​= ​0.081; see Table 4).

**Table 4:** Mean levels and group comparisons of plasma proinflammatory cytokines at Visit 1 and Visit 2.

Proinflammatory Cytokine	Control		CD		UC		*p*- value
	Visit 1	Visit 2	Visit 1	Visit 2	Visit 1	Visit 2	
IL-6 (pg/ml)	0.87 ​± ​0.09	1.32 ​± ​0.26	2.14 ​± ​0.41^Ϯ^	4.06 ​± ​0.91^¥^	2.77 ​± ​1.75	4.05 ​± ​3.27	<0.001∗∗∗
IL-8 (pg/ml)	4.49 ​± ​0.32	7.81 ​± ​0.64	6.55 ​± ​0.83	14.08 ​± ​5.08	11.32 ​± ​6.08	4.99 ​± ​2.05^#^	0.068
TNF-α (pg/ml)	4.52 ​± ​0.24	5.13 ​± ​0.2	5.52 ​± ​0.56	6.95 ​± ​0.77	3.64 ​± ​0.83^$^	1.91 ​± ​0.64^*¥*#^	0.001∗∗

IL, interleukin; CD, Crohn’s disease; UC, ulcerative colitis. p-value represents group effect from repeated measures ANOVA; ∗∗p ​< ​0.01; ∗∗∗p ​< ​0.001. ^Ϯ^p<0.001 vs control Visit 1; ^¥^p ​< ​0.001 vs control Visit 2; ^#^p ​< ​0.001 vs CD Visit 2; ^$^p ​< ​0.001 vs CD Visit 1. Data are mean ​± ​S.E.M.

#### 3.4.2 IL-8 levels are lower in patients with ulcerative colitis at visit 2

Plasma levels of IL-8 at visit 2 were significantly lower in patients with UC when compared with patients with CD (*P* ​= ​0.002) and healthy control participants (*P* ​= ​0.014, see Table 4).

#### 3.4.3 TNF-α levels are lower in patients with ulcerative colitis at visit 1 and 2

Analysis of levels of TNF-α across visit 1 and 2 showed a significant main effect of group (F (2, 49) ​= ​19.995; *P* ​< ​0.001, η_p_^2^ ​= ​0.449), with patients with UC having significantly lower levels of TNF-α when compared to both patients with CD (*P* ​< ​0.001) and healthy control participants (*P* ​< ​0.001; see Table 4).

### 3.5 Plasma tryptophan, kynurenine & Kyn:Trp ratio

#### 3.5.1 Tryptophan levels are not different between patients and healthy controls

Plasma tryptophan levels did not significantly differ between groups at visit 1 or 2 (all *P* ​> ​0.05; Table 5).

**Table 5:** Mean levels and group comparisons of plasma tryptophan, kynurenine and kynurenine:tryptophan ratio at Visit 1 and Visit 2.

	Control		CD		UC		*P*-value
	Visit 1	Visit 2	Visit 1	Visit 2	Visit 1	Visit 2	
Tryptophan	11923 ​± ​426.35	12122.91 ​± ​442.79	11002.09 ​± ​588.32	12563.59 ​± ​1003.27^*Ϯ*^	11452.74 ​± ​1226.7	9984.83 ​± ​828.9	0.413
Kynurenine	522.41 ​± ​23.90	527.67 ​± ​23.98	553.35 ​± ​43.03	645.16 ​± ​44.36	642.2 ​± ​68.22	542.16 ​± ​58.9	0.177
Kyn:Trp ratio	0.04 ​± ​0.0	0.04 ​± ​0.0	0.05 ​± ​0.0	0.06 ​± ​0.01 ^*#*^	0.06 ​± ​0.00 ^*¥*^	0.05 ​± ​0.1	0.023∗

CD, Crohn’s disease; UC, ulcerative colitis. p-value represents main group effect from repeated measures ANOVA across Visit 1 and 2; ∗p ​< ​0.05. ^Ϯ^p<0.05 vs control Visit 2; ^¥^p ​< ​0.05 vs control Visit 1; ^#^p ​< ​0.001 vs control Visit 2. Data are mean ​± ​S.E.M.

#### 3.5.2 Kynurenine levels are significantly elevated in patients with Crohn’s disease at visit 2

A main effect of group for plasma kynurenine levels was evident at visit 2 (see Table 5) with patients with CD having significantly elevated levels when compared to healthy control participants (*P* ​= ​0.039) but not compared to patients with UC (*P* ​= ​0.286).

#### 3.5.3 Kynurenine: tryptophan (Kyn:Trp) ratio is significantly elevated in patients with ulcerative colitis at visit 1 and patients with Crohn’s disease at visit 2

Analysis of the plasma Kyn:Trp ratio across visit 1 and 2 showed a significant main effect of group (see Table 5), with patients with CD having significantly elevated levels when compared to healthy control participants (*P* ​= ​0.03), but not when compared with patients with UC (*P* ​= ​0.997).

### 3.6 Correlational analysis

Correlational analysis revealed no significant relationships between attentional (*Stroop Test*) or visuospatial memory performance (PAL *total errors*), and biochemical measures, self-report psychological or clinical measures (see Table 6).

**Table 6:** Summary of correlations between averaged Visit 1 and 2 values for selective attention and response inhibition (Stroop Interference Effect), visuospatial memory performance (PAL Total Errors), physiological markers, anxiety, depression and disease activity.

	Crohn’s Disease (CD) Group	
	Stroop Interference Effect (Mean of visit 1 & 2)	PAL Total Errors (Mean of visit 1 & 2)
Measure (Mean of visit 1 & 2)		
*IL-6*	-.186	-.032
*IL-8*	-.207	-.043
*TNF-a*	.368	.154
L-Tryptophan	-.304	-.086
L-Kynurenine	-.075	-.243
*Kyn:Trp Ratio*	-.107	-.171
*CAR (AUCg)*	.358	.154
HADS-A (*Anxiety*)	-.022	.038
HADS-D (*Depression*)	.132	.221
PHQ-9 (*Depression*)	-.126	.005
*Disease Activity (HBI)*	-.011	.051
*Disease Duration*	.215	-.022
*WAIS IQ*	-.082	-.490

PAL, Paired Associate Learning; IL, interlukin; TNF-a, tumor necrosis factor-alpha; Trp:Kyn; kynurenine:tryptophan ratio; HADS-A/D, Hospital Anxiety and Depression Scale- Anxiety/Depression; HBI, Harvey Bradshaw Index; WAIS-R, Wechsler Adult Intelligence Scale-Revised.

## 4 Discussion

Our primary aim was to prospectively assess cognitive performance in patients with IBD in clinical remission in comparison to healthy control participants, with a focus on elaborating on previous findings indicating a selective attention and response inhibition deficit in these patients with CD (Kennedy et al., 2014). To our knowledge, this is the first study to prospectively assess cognitive performance in CD patients and to examine the role of a range of biomarkers that may impact on cognition. In agreement with previous findings (Kennedy et al., 2014), we found that when patients with CD were prospectively followed over a 6 month period, they displayed a persistent deficit in attentional performance. Interestingly, patients with UC did not display a similar deficit. In addition to impaired attentional performance, patients with CD exhibited significantly impaired visuospatial memory performance across visits.

Reports of cognitive impairment in IBD have been inconsistent (Berrill et al., 2013; Dancey et al., 2009). A number of reasons may be proposed to explain these disparate findings including differing levels of disease activity and medication use in patients, sample characteristics and approach to subject matching (e.g. by age, IQ or years of education), or, indeed, differences in the psychometric properties of the cognitive assessments which were employed. However, a recent structural magnetic resonance imaging study reported that patients with CD in clinical remission have reduced grey matter volume in regions of the dorsolateral prefrontal cortex and anterior midcingulate cortex (aMCC (Agostini et al., 2013). Functional alterations in the ACC and the right inferior frontal gyrus have also been reported (Petruo et al., 2017) in addition to alterations in brain white matter microstructural properties (Hou et al., 2020). Regions of the anterior cingulate cortex (e.g. aMCC, posterior MCC) are heavily involved in attentional decision-making tasks such as the Stroop (Bush, 2009; Enriquez-Geppert et al., 2013). As such, our findings compliment these neuroimaging findings and raise the possibility that the structural brain changes which are apparent in patients with CD in clinical remission are associated with functional impairments in ACC-mediated cognitive performance.

Interestingly, attention and memory impairments have also been reported in pediatric patients with IBD (Piasecki et al., 2017). However, additional studies in which both structural neuroimaging and cognitive testing are performed in the same cohort of patients are needed to verify this hypothesis. These observations also need to be integrated with the recent reports of impaired neurocognitive and psychomotor function across areas of convergent thinking, perceptive abilities and sophisticated operative thinking (Tadin Hadjina et al., 2019) as well as cognitive inflexibility (Petruo et al., 2017). Emotional processing biases have also been reported to contribute to co-morbid depression among people with IBD (Wilkinson et al., 2019) while anxiety symptoms were associated with slower processing speed, lower verbal learning, and lower working memory performance (Whitehouse et al., 2019).

Despite not finding any relationships between the biomarkers measured and cognitive performance in patients with CD, there is substantial evidence that elevated proinflammatory cytokines (Dantzer, 2009; Quan and Banks, 2007), circulating cortisol (Kennedy et al.) and kynurenine metabolism (Kennedy et al., 2015; Stone and Darlington, 2013) can modulate CNS function and cognitive performance. As such, we cannot rule out this reflects the difficulty in using correlational techniques to identify complex non-linear neurobiological relationships. Conversely, it may reflect a need to measure relationships between these biochemical parameters and cognition over much longer periods and at multiple time-points to identify meaningful covariations.

Future studies that are designed to elucidate the impact of disease activity on cognitive performance are needed. We did not find any relationships between cognitive performance and disease activity. However, we did not measure additional GI symptoms such as pain or bloating which may well impinge on cognitive performance. Therefore, future studies employing a well validated and temporally specific GI symptom assessment in relation to cognitive performance in IBD are needed. In addition, our groups differed with respect to gender, and although the effect of gender on cognitive performance tends to be small and inconsistent (Weiss et al., 2003), it will be important for future investigations to examine the role that gender plays in mediating the impact of pathogenic processes impacting on cognitive performance in IBD. Moreover, such studies should aim to delineate the role of key factors such as menstruation status and age in impaired cognitive performance in IBD.

In conclusion, patients with CD in clinical remission, followed prospectively, exhibit a consistent impairment in attentional performance and visuospatial memory on the PAL test. Accumulating evidence supports the view that patients with IBD should be monitored for psychological well-being (Gracie et al., 2018). Moreover, evidence for the efficacy of antidepressants is now emerging in IBD (Mikocka-Walus et al., 2020). Our findings, taken with previous reports, extend the psychological component of CD to the cognitive domain and indicate that altered attention may be related to neurobiological changes in the function of ACC brain regions. This has significant clinical implications by raising the issue of how this deficit impacts on the functional capacity of patients, their experience of illness and their quality of life. Thus, future interventional studies should aim to identify which therapeutic strategies alleviate not only the inflammatory GI symptoms, but also co-morbid cognitive impairment in CD.

## Author contributions

Drafted the manuscript; TGD, JFC, FS, EMMQ, JAG, GC and PJK; provided study concept and design: TGD, JFC, FS, EMMQ, JAG, and GC; contributed to interpretation of the data and statistical analysis: TGD, JFC, FS, EMMQ, JAG, GC and PJK; coordinated acquisition of data and study supervision: PJK; approved this final draft for submission: PJK, GC, JAG, FS, EMMQ, JFC and TGD.

## Declaration of competing interest

APC Microbiome Ireland has conducted studies in collaboration with several companies, including GSK, Pfizer, Cremo, Suntory, Wyeth, Mead Johnson, Nutricia, 4D Pharma, and DuPont. T. G. Dinan has been an invited speaker at meetings organized by Servier, Lundbeck, Janssen, and AstraZeneca and has received research funding from Mead Johnson, Cremo, Suntory Wellness, Nutricia, and 4D Pharma. J. F. Cryan has been an invited speaker at meetings organized by Mead Johnson, Yakult, Alkermes, and Janssen and has received research funding from Mead Johnson, Cremo, Suntory Wellness, Nutricia, DuPont, and 4D Pharma. G Clarke has been an invited speaker at meetings organized by Janssen and is receipt of research funding from Pharmavite. The authors are not aware of any affiliations, memberships, funding, or financial holdings that might be perceived as affecting the objectivity of this report.
